# Estimating the Health Effects of Greenhouse Gas Mitigation Strategies: Addressing Parametric, Model, and Valuation Challenges

**DOI:** 10.1289/ehp.1306744

**Published:** 2014-02-28

**Authors:** Justin V. Remais, Jeremy J. Hess, Kristie L. Ebi, Anil Markandya, John M. Balbus, Paul Wilkinson, Andy Haines, Zaid Chalabi

**Affiliations:** 1 Department of Environmental Health, Rollins School of Public Health, Emory University, Atlanta, Georgia, USA; 2 Department of Emergency Medicine, School of Medicine, Emory University, Atlanta, Georgia, USA; 3 ClimAdapt, LLC, Los Altos, California, USA; 4 Ikerbasque, Basque Centre for Climate Change, Bilbão, Spain; 5 National Institute of Environmental Health Sciences, National Institutes of Health, Department of Health and Human Services, Bethesda, Maryland, USA; 6 Department of Social and Environmental Health Research, Faculty of Public Health and Policy, London School of Hygiene and Tropical Medicine, United Kingdom

## Abstract

Background: Policy decisions regarding climate change mitigation are increasingly incorporating the beneficial and adverse health impacts of greenhouse gas emission reduction strategies. Studies of such co-benefits and co-harms involve modeling approaches requiring a range of analytic decisions that affect the model output.

Objective: Our objective was to assess analytic decisions regarding model framework, structure, choice of parameters, and handling of uncertainty when modeling health co-benefits, and to make recommendations for improvements that could increase policy uptake.

Methods: We describe the assumptions and analytic decisions underlying models of mitigation co-benefits, examining their effects on modeling outputs, and consider tools for quantifying uncertainty.

Discussion: There is considerable variation in approaches to valuation metrics, discounting methods, uncertainty characterization and propagation, and assessment of low-probability/high-impact events. There is also variable inclusion of adverse impacts of mitigation policies, and limited extension of modeling domains to include implementation considerations. Going forward, co-benefits modeling efforts should be carried out in collaboration with policy makers; these efforts should include the full range of positive and negative impacts and critical uncertainties, as well as a range of discount rates, and should explicitly characterize uncertainty. We make recommendations to improve the rigor and consistency of modeling of health co-benefits.

Conclusion: Modeling health co-benefits requires systematic consideration of the suitability of model assumptions, of what should be included and excluded from the model framework, and how uncertainty should be treated. Increased attention to these and other analytic decisions has the potential to increase the policy relevance and application of co-benefits modeling studies, potentially helping policy makers to maximize mitigation potential while simultaneously improving health.

Citation: Remais JV, Hess JJ, Ebi KL, Markandya A, Balbus JM, Wilkinson P, Haines A, Chalabi Z. 2014. Estimating the health effects of greenhouse gas mitigation strategies: addressing parametric, model, and valuation challenges. Environ Health Perspect 122:447–455; http://dx.doi.org/10.1289/ehp.1306744

## Introduction

Climate change poses one of this century’s most significant public health challenges (Chan 2009). There is growing recognition that strategies to reduce greenhouse gas (GHG) and climate-active aerosol emissions (“mitigation” strategies) will affect numerous upstream drivers of public health, including indoor and outdoor air pollution, water security and quality, food security and quality, and physical activity, with the potential for beneficial and adverse impacts (Table 1; Haines et al. 2009; Little and Jackson 2010; Newmark et al. 2010).

**Table 1:** Summary of major health drivers and outcomes modified by select mitigation strategies.

Sector/mitigation strategy	Health drivers	Health and related outcomes potentially affected
Energy (Burtraw etal. 2003; Markandya etal. 2009)
Reduce fossil fuel combustion	Reduce conventional air pollutants: particulate matter, ozone, nitrogen oxides, volatile organic compounds	Cardiovascular morbidity and mortality; asthma and other respiratory diseases; developmental disorders; improved crop survival and productivity
Increase production of some types of biofuels	Increase food prices and lower availability depending on whether they compete directly with food crops	Food insecurity; malnutrition
Carbon capture and sequestration	Groundwater availability and quality; contamination with metals and minerals, sudden carbon dioxide/hydrogen sulfide releases	Various related to specific contaminants
Transportation (Cifuentes etal. 2001; Maizlish etal. 2013; Shindell etal. 2011; Woodcock etal. 2013)
Improve fuel economy; increase adoption of electric and other noncombustion engines; tighter on-road vehicle emissions standards	Reduce conventional air pollutants	Cardiovascular morbidity and mortality; asthma and other respiratory diseases;
Increase access and convenience of active modes of transportation, including walking, cycling, and publictransit	Reduce conventional air pollutants	Cardiovascular morbidity and mortality; asthma and other respiratory diseases; developmental disorders
	Increase physical activity levels	Cardiovascular morbidity and mortality; obesity and diabetes risk; risk of certain cancers; risk of dementia, depression, injury
Agriculture (Friel etal. 2009; McMichael etal. 2007)
Reduce ruminant livestock production; capture methane emissions	Reduce ozone air pollution	Cardiovascular and respiratory morbidity and mortality
	Reduce consumption of animal products with high levels of saturated fat; reduce red and processed meat consumption; increase consumption of unsaturated fats ofvegetable origin and of fruit and vegetables	Cardiovascular morbidity and mortality; risk of certain cancers including large bowel cancer
Land use in built environment (Younger etal. 2008)
Increase green space and parks in built environment; increase shading and vegetation along roads	Increase physical activity; reduce excessive temperature exposure	Cardiovascular risk; some cancer risks; mental health

Importantly, many mitigation-related health impacts accrue sooner than the impacts projected from climate change. Studies published in the *Lancet* in 2009 highlighted this, suggesting significant net health benefits across several mitigation strategies and settings (e.g., Haines et al. 2009). Studies in this series used modeling to estimate the differences in, and magnitude of, health co-benefits of mitigation actions in various sectors, as well as discussing the potential for adverse health impacts, or co-harms. Subsequent analyses in the United States extended these findings (Grabow et al. 2012; Maizlish et al. 2013).

Studies estimating the ancillary health effects of mitigation strategies (termed “co-benefits” from here forward, with the acknowledgment that co-harms also may result) use a range of modeling approaches, drawing expertise from public health, agriculture, environmental sciences, urban planning, and other disciplines to generate policy-relevant outputs. We reviewed several specific issues with modeling co-benefits of mitigation strategies, including those related to model framework, structure, and choice of parameters, and the implications of these for policy uptake. Some of these issues are common to other types of modeling, so our discussion could be applied to similar concerns arising in the development of health impact assessments (European Centre for Health Policy 1999; Kemm 2007) and the modeling of certain climate change adaptation activities, which also have co-benefits and co-harms (Cheng and Berry 2013). We focused specifically on mitigation co-benefits modeling, however, for several reasons: First, all co-benefits modeling of climate change mitigation policies necessarily requires attention to these issues, whereas not all health impact assessment efforts, or efforts to quantify ancillary impacts of adaptation strategies, do. Second, GHG emission reduction policies can influence a range of major risk factors that contribute substantially to global disease burden, whereas climate change adaptation strategies result in health co-benefits predominantly by increasing resilience to existing climate variability. Third, the field of health impact assessment studies is much broader and would require a wider-ranging discussion. And fourth, to date there has not been a systematic consideration of the methodological issues related to modeling health co-benefits of climate change mitigation policies.

**Figure 1:**
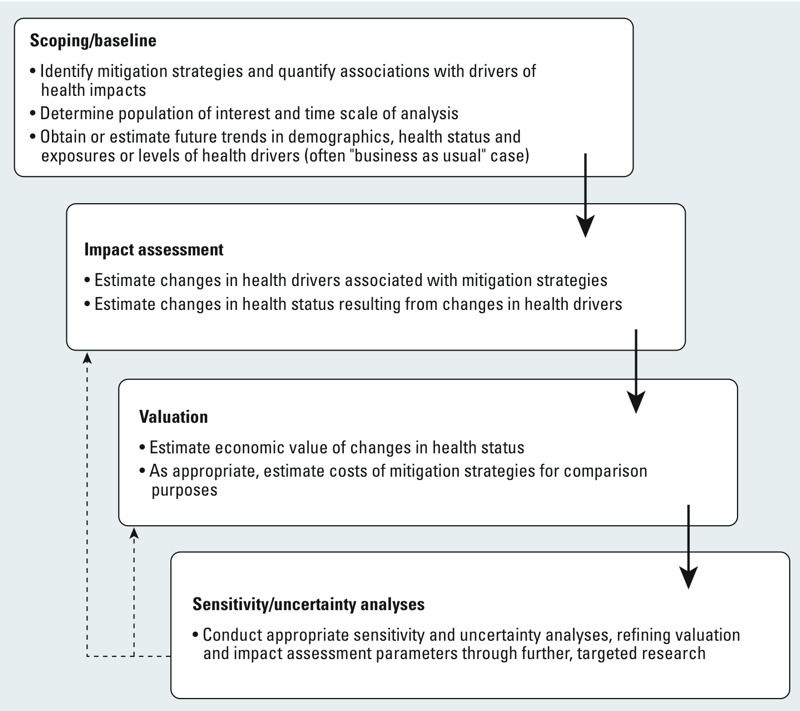
Model of health effects of mitigation showing scoping activities that define the initial and boundary conditions of the analysis; impact assessment; valuation procedures; and sensitivity and uncertainty analyses, the results of which can be used to further refine impact assessment and valuation analyses (dashed lines).

Modeling of co-benefits generally takes the basic approach shown in Figure 1, employing a wide variety of methods such as comparative risk assessment (Smith and Haigler 2008), complex mechanistic components (such as those describing building physics, e.g., Wilkinson et al. 2009); and macroeconomic, technological, and behavioral models (National Research Council 2010). The range of modeling approaches commonly used is detailed in Supplemental Material, Table S1; the table also includes central estimates of health co-benefits reported by selected studies.

Several overlapping challenges are common to co-benefits modeling studies [Bell et al. 2008; Haines et al. 2009; HEI (Health Effects Institute) International Scientific Oversight Committee 2010; Matus et al. 2008; Patz et al. 2008; Smith and Haigler 2008], including the following:

Modeling the time course of strategies that are phased in over time, and the resulting time-varying levels of exposures to health drivers;
Taking into account the varying lag times between changes in exposure and changes in health outcomes according to the health outcome concerned;
Incomplete methods for quantifying and conveying the degree and sources of uncertainty associated with the modeling outputs;
Debate over key parameters, such as discount rates and terms involved in the economic valuation of health outcomes; and
Estimating future economic development pathways and GHG emissions, and projecting trends in demographics, health status, and levels of exposures to health drivers over the relevant time course.

This review is an initial effort to address some of these challenges, with a focus on modeling issues (time course of exposures and impacts; uncertainty; and low-probability/high-impact effects) and issues affecting relevance (discount rate selection, decision analysis, and inclusion of factors affecting policy uptake and system dynamics). We conclude with recommendations to advance the rigor and consistency of co-benefits modeling.

## Key Modeling Issues

Health co-benefits models typically begin with a mapping exercise that proceeds to a more formal mathematical model describing relationships between model components and outcomes of interest. This process may involve identification of specific indicators of health impacts. A number of different frameworks are available (e.g., Hambling et al. 2011), and the relationships identified in the mapping process can be formally quantified and assessed using a variety of strategies.

*Initial mapping to model construction*. Modeling can be used to answer a specific set of policy questions regarding the health impacts of particular mitigation options. An important initial step is developing a conceptual framework linking the mitigation policy to specific public health drivers in the near- and mid-term over which beneficial health impacts accrue. Modeling efforts begin with description of the system boundaries, major associations between different model components, outcome indicators and their metrics, and definition of the counterfactuals (e.g., “business as usual”) used for comparison. For instance, in estimating the impact of introducing low-emission cookstoves in India on health impacts of household air pollution, the initial conceptual map included population growth and demographics, proportion of the population with low-emission cookstoves, major health outcomes associated with elevated levels of household air pollution, and historical experience implementing national cookstove interventions, but not the potential effects on household income (Wilkinson et al. 2009).

The models constructed from these mapping exercises should capture the key associations between model components and the outcomes of interest within the scale and scope of the project. Unfortunately, not all relationships are well understood, and not all parameters are well studied. For instance, there are questions about the mitigation potential of cookstove interventions because stove emissions can affect climate negatively or positively (Wilkinson et al. 2009). Likewise, poor maintenance of household energy interventions such as anaerobic digesters can lead to direct emissions of potent GHGs into the atmosphere (Dhingra et al. 2011), potentially limiting their long-term performance. Although such uncertainty does not affect the resulting estimates of health impacts of a mitigation strategy, it does affect the confidence in estimates of efficacy of the mitigation strategy relative to other options (Haines et al. 2009). Modelers must decide what to include and how to define the range of input parameters based on the best available evidence.

*Modeling complex, time-varying exposures and impacts*. Several key time-varying elements of mitigation policies must be made explicit, such as the time course for intervention implementation (e.g., low-emission cookstoves) and associated exposure changes (e.g., reductions in household air pollution). Mitigation activities may be represented in models as enacted instantaneously, in steps, or gradually phased in, although most integrated assessment models assume instantaneous and perfect implementation (first-best worlds). Most co-benefit models consider step changes in mitigation interventions (Cifuentes et al. 2001; Maizlish et al. 2013; Woodcock et al. 2013). Ideally, models should employ a time course empirically based on analogous interventions (Wilkinson et al. 2009). Similarly, exposures should be modeled to reflect those temporal characteristics most strongly associated with health outcomes—for example, peak levels are most relevant for some hazards, cumulative and long-term exposures for others (Lin et al. 2008; Murray et al. 2003; Robins and Hernan 2009). The dynamic response between disease and exposure must also be considered, requiring an accounting of cumulative exposures and associated morbidity and mortality among an age-stratified cohort over time (Matus et al. 2008). Table 2 shows the approximate time lags over which health co-benefits are likely to accrue for the strategies explored in recent co-benefit analyses (Friel et al. 2009; Jarrett et al. 2012; Wilkinson et al. 2009; Woodcock et al. 2013).

**Table 2:** Time lags over which the health co-benefits accrue for the mitigation strategies explored in recent health effects of mitigation modeling studies.^*a*^

Health outcome	Likely time lag for health co‑benefits
Reductions in sudden cardiac death risk due to reduced air pollution	Days to weeks
Reduction in acute respiratory infections in children due to reduced air pollution	Weeks and months
Reduction in chronic obstructive pulmonary disease (COPD) exacerbations	Weeks and months
Reduction in ischemic heart disease events due to partial substitution of animal source saturated fat consumption by polyunsaturated fats of plant origin	Years
Reduction in type 2 diabetes due to change in physical activity	Years
Reduction in depression due to change in physical activity	Years
Reduction in breast and colon cancer incidence due to change in physical activity	Years
Reduction in COPD prevalence due to reduced air pollution	Decades
**a**Friel et al. (2009); Jarrett et al. (2012); Wilkinson et al. (2009); Woodcock et al. (2013).	

Numerous methods are available to incorporate time-varying exposures and associated time-varying health effects when appropriate, including comparative risk assessment approaches (Lin et al. 2008; Murray et al. 2003), modification of the standard static Cox proportional hazard model (Haneuse et al. 2007), and functional approximation methods that associate health outcomes with exposure history (Bandeen-Roche et al. 1999). As an alternative, co-benefits studies can use time functions not directly derived from epidemiological studies that are parameterized to simulate the time lag in health effects in response to changes in exposure. For example, Jarrett et al. (2012) used sigmoid lag functions to simulate delays in the response of depression, ischemic heart disease, and other effects to changes in exposure to physical activity.

Estimating adverse effects of mitigation strategies. The validity of a modeling analysis depends partly on inclusion of all relevant pathways among mitigation strategies, consequent exposures, and outcomes of interest. This requires including pathways that increase risk (co-harms) or decrease it (co-benefits). Potential co-harms of various mitigation strategies include reduced affordability of food leading to poor nutrition [if, for example, pastoralists in poor countries have to reduce their consumption of animal products (Friel et al. 2009)]; rising energy costs pushing the poor toward low-quality biomass fuels (Markandya et al. 2009); and increases in air pollution from combustion of biofuels (Jacobson 2007).

An example of an adverse impact with a relatively simple causal pathway is increased pedestrian and cyclist exposure to road traffic injuries resulting from an increase in active transport (DiGuiseppi et al. 1997; Jarrett et al. 2012; Woodcock et al. 2009). In one analysis, estimated increases in morbidity and mortality from pedestrian and cyclist road traffic injuries in London (UK) were more than offset by decreases in disability-adjusted life years (DALYs) lost from physical inactivity and to a lesser extent air pollution (Woodcock et al. 2009), a finding reinforced by Lindsay et al. (2011). More complex, indirect pathways can also yield adverse impacts—for example, switching some agricultural production from food to biofuel feedstocks can have complex, recursive macroeconomic effects including shifts in prices of various food staples (Chakravorty et al. 2009). In 2007, for instance, expanded biofuels production was estimated to be responsible for approximately 30% of the rapid rise in grain prices (Rosegrant 2008). Such price increases, along with other economic shocks, increase undernutrition (Bloem et al. 2010; Friel et al. 2009), a major risk factor for mortality of children < 5 years of age (Black et al. 2008). One analysis found that such dynamics likely increased child mortality in East and Southeast Asia in 2007 (Bhutta et al. 2008; Christian 2010). Large uncertainties exist, including the complex relationships among supply, demand, and global food prices (Mitchell 2008); in regional resilience to price spikes (Webb 2010); and in other drivers for the multiple health end points of undernutrition (Black et al. 2008). Despite these difficulties, nutrition-mediated health effects of some biofuel policies serve as a good example of a tractable co-harms estimation problem that could be used to inform future mitigation decisions (Bloem et al. 2010; Christian 2010; Friel et al. 2009).

Low-probability events with highly adverse impacts. Certain mitigation technologies are associated with low-probability/high-impact co-harms, such as severe nuclear power plant accidents, catastrophic failures of so-called “mega-dams,” and leaks from carbon capture and storage (Bickel and Friedrich 2005; Markandya et al. 2009). This class of adverse impacts is challenging to estimate: low probability high impact exposures are highly uncertain and episodic, so deterministic exposure functions cannot be directly applied. Event (i.e., accident) data for certain mitigation options are sparse, making alternative analytical approaches, such as estimating expected damage, difficult (e.g., Ha-Duong and Loisel 2010). Importantly, when the expected harms of these risks are quantified, estimated impacts can be considerably smaller than public perceptions of these risks (Krupnick et al. 1993). Incorporating risk perception heuristics—in which the public views risks associated with these events as more problematic than more routine events with the same expected value (Bier et al. 1999)—into co-benefits modeling is an important frontier to explore.

*Methods for the treatment of uncertainty*. Uncertainties are inherent to modeling studies and permeate complex policy decisions such as those surrounding climate change mitigation. Uncertainties in modeling health co-benefits include *a*) simulating the spatial and temporal changes in health-relevant exposures; *b*) determining the time response of the health effects due to exposure changes; *c*) comparing alternative mitigation interventions in terms of their health effects across populations and time scales; and *d*) establishing the assumed time course of future disease-specific burdens in the absence of mitigation.

There has been much discourse on dealing with uncertainty, particularly with respect to the integrated assessment models used to evaluate mitigation policies, that is relevant for co-benefits modeling (Mearns 2010; Rotmans and Van Asselt 2001a, 2001b; Visser et al. 2000; Webster et al. 2003). Co-benefits studies often take a simplistic, one-dimensional approach to propagating the multiple sources of uncertainty (Schneider and Kuntz-Duriseti 2002). Uncertainties are cascaded sequentially through model components starting with “upstream” drivers (e.g., mitigation options, emissions, carbon cycle response, and global climate sensitivity) and then “downstream” to local climate change, exposures, and health impacts. Socioeconomic change, as an example, contributes significant downstream uncertainty (Arnell et al. 2004). In some circumstances the combined uncertainty, particularly over the long term, makes it difficult to determine the balance of costs, co-benefits, and co-harms, but additional methods can help narrow estimates substantially, particularly in the near term. The following sections summarize several quantitative approaches. Overcoming challenges in integrating quantitative and nonquantitative approaches to uncertainty characterization is also very important.

Uncertainty propagation through models. Model uncertainty can be classified as structural or parametric (Refsgaard et al. 2006; Tebaldi and Knutti 2007). Structural uncertainty refers to uncertainty in the constitution of the model, such as the configuration of the air dispersion Gaussian model, the makeup of the exposure pathways (e.g., inhalation, ingestion), and the types of exposure–response relationships (e.g., linear, threshold-linear, nonlinear). Structural uncertainty also results from assumptions and simplifications used to construct the health model (Bojke et al. 2009). Parametric uncertainty, on the other hand, relates to uncertainty in the model’s parameters, conditional on a specific structure, such as uncertainties in the threshold and slope of a threshold-linear exposure–response relationship, or the indoor/outdoor concentration ratio for PM_2.5_ (particulate matter with aerodynamic diameter ≤ 2.5 μm). Such types of uncertainty permeate science and conventional epidemiological research, such as in the relationship between an energy efficiency intervention and exposure to household air pollutants (Table 3 shows several examples).

**Table 3:** The types of downstream uncertainties in recent health effects of mitigation modeling studies.^*a*^

Sector	Parametric uncertainties	Structural uncertainties
Household energy
Specification of mitigation scenarios	Average value of reduction in GHG emissions due to insulation improvements	Feasible transitions from household fossil fuel combustion to electricity
Estimating exposures	Values of the parameters of building physics model	Occupant behavior and increased consumption of resources given higher end-user efficiency
Estimating health impacts	Values of the pollutants’ relative risk coefficients	Pollutants to consider in the assessment
Urban land transport
Specification of mitigation scenarios	Percentage increase in the level of active travel (walking and cycling)	Nonlinear “safety in numbers” effect of increase in proportion of cyclists on rates of cyclist injuries; different future “active travel visions”
Estimating exposures	The values of the parameters of the emission–dispersion air pollution model	Reduction of emissions from transport in London are representative for other European cities; reduction in transport emissions results in proportional reduction in particulate matter
Estimating health impacts	The values of the physical activity–disease relative risk coefficients	Diseases affected by physical activity; linear versus nonlinear relationships between physical activity and health outcomes
Food and agriculture
Specification of mitigation scenarios	Percentage reduction in livestock production by 2030	Contribution of different livestock to greenhouse emissions and different assumptions about feedstocks
Estimating exposures	Percentage reduction in intake of saturated fat	Full replacement of saturated fats with unsaturated fats
Estimating health impacts	Saturated fat-ischemic heart disease mortality relative risk coefficient	Exposure–health outcome pathways
^***a***^Friel et al. (2009); Maizlish et al. (2013); Wilkinson et al. (2009); Woodcock et al. (2013); these uncertainties are naturally not unique to co-benefits modeling.		

Although there is no single best way to characterize uncertainty in an analysis, there is a need for consistency and transparency in handling it. Indeed, many of the methods used for handling uncertainty in complex environmental models can be used in this context (Rao 2005; Refsgaard et al. 2007), as can deterministic and stochastic techniques from health impact models (Lopez et al. 2006). Several unique uncertainty issues arise in co-benefit analyses, such as the uncertainty in future projections over the time horizon of analysis of disease-specific burdens in the absence of mitigation. These projections are the baseline against which burdens with mitigation are compared, and thus represent a primary source of uncertainty. The current disease burden is often adopted as the baseline; but this is rarely appropriate because development will occur and bring with it technology and other changes that will alter disease burdens, such as the ongoing, rapid increases in the burden of noncommunicable diseases in low- and middle-income countries (Remais et al. 2013).

Characterizing structural uncertainty. There are two main approaches for characterizing structural uncertainty in co-benefits modeling. The first simulates different model structures and then combines their outputs deterministically (e.g., Knutti et al. 2010); the second does the same but combines the outputs probabilistically (e.g., Min et al. 2007). The first approach is easier to implement, particularly for co-benefit analyses with a small number of alternative model structures. The output is either a series of single co-benefit projections (one for each structure or combinations of structures), or a sum of outputs weighted by the confidence in the model structure used to generate each. The second approach uses Bayesian model averaging to produce a weighted probability density function. This approach is useful when there are many alternative model structures to consider, but may not be feasible when the computational time to run each alternative model structure is high.

Structural uncertainties can have large impacts on estimated health effects of mitigation. For instance, in the Woodcock et al. (2009) analysis of the health effects of increased physical activity resulting from transport-related mitigation strategies, uncertainty in the physical activity exposure–response relationship (e.g., linear vs. square root) led to more than a doubling of the estimated health effects as measured by premature deaths or DALYs lost. To characterize the influence of structural sources of uncertainty, alternative model structures (i.e., functional forms) can be used to represent the exposure–response relationship, providing an estimate of the uncertainty in health effects as a function of structural choices.

Characterizing parametric uncertainty. Parametric uncertainty can arise in situations where there is limited information on the nominal or central value of a model parameter. For instance, in assessing the health co-benefits of mitigation in México City, México, Cifuentes et al. (2001) calculated the central estimate of the number of premature deaths avoided as 29,055 in the period 2000–2020. The authors used an estimate of the uncertainty in the relative risk in mortality for a 10-μg m^–3^ change in PM_10_ (PM with aerodynamic diameter ≤ 10 μm) concentration to calculate the 95% CI of premature deaths avoided (9,265, 56,293). An alternative approach, particularly useful when an estimate of the variance of parameter is unavailable, is to characterize the uncertainty in the relative risk as an interval (i.e., the parameter’s value can be anywhere between a lower and upper bound) and compute an associated interval of model output (De Figueiredo and Stolfi 2004). Such parameter bounds can be elicited from expert opinion, literature reviews, or model simulations.

Finally, stochastic approaches are also available in which a probabilistic sensitivity analysis is carried out with parameter values drawn randomly from the respective parameter spaces. In this case, Monte Carlo (MC) simulation or Latin hypercube sampling (LHS) was used to repeatedly sample the parameter space, generating a distribution of model outputs. These methods are widely used when the uncertainty in parameters can be expressed as probability density functions (Helton et al. 2005). LHS is a stratified version of MC sampling that for the same number of samples is more likely to reproduce faithfully the probability density function than MC sampling; MC sampling, on the other hand, is easier to implement (McKay et al. 1979). Recent advances in dynamic sensitivity analysis (Wu et al. 2013) may offer promise for co-benefits analyses where complex dynamics result from the coupling of shifting time courses of mitigation phase-in, time-varying exposures, and varying lag times over which health impacts evolve.

Propagating uncertainties. Uncertainty propagation through a series of model components should be consistent with fundamental principles of error propagation, with proper linking of submodel outputs and inputs (Mekid and Vaja 2008). Yet standard error propagation can quickly become infeasible for large, multipart models. For example, in calculating the health co-benefits of GHG mitigation in the electricity sector in the United States, Burtraw et al. (2003) combined two large-scale models in which the output of one model fed into the input of the other. The first model simulated electricity demand, generation, consumption, and emissions of air pollutants; the second model took the emissions from the first and calculated the associated health impacts. Each model comprised a number of complex submodels (e.g., pollutant transport, dose response), and, although this was not attempted, only a limited propagation of uncertainties through this long chain of models and submodels would have been possible. Even when quantitative uncertainty propagation is feasible, additional information can be gained from qualitative approaches, such as storylines, that can represent uncertainties associated with different futures (e.g., Arnell et al. 2004).

Using value-of-information (VOI) analysis to identify key uncertainties that can be reduced. Given the diversity of uncertain parameters in health co-benefits modeling and the infeasibility of investigating all uncertain parameters, there is a need to determine the parameters whose uncertainty would be most easily and strategically reduced through additional research. Experts can use a VOI analysis to determine which new data will most likely yield more precise estimates. VOI analysis determines the return, or the payoff in terms of making better decisions, of collecting additional information (Yokota and Thompson 2004). VOI has been used to identify research priorities in climate change research (Rabl and Van der Zwaan 2009), although not yet to improve parameterization of models used to estimate health co-benefits of mitigation policies. Reduced parametric uncertainty can help decision makers avoid costly errors, and future co-benefits analyses may choose to express the expected return of investing in improved parameter estimates in monetary terms (Coyle and Oakley 2008).

## Addressing Key Science Policy and Decision Support Issues

Co-benefits models are generally intended to inform the policy-making process, including modeling carried out in response to a specific policy question under consideration by a particular governing body. Rising interest in the links between climate change mitigation and public health will increase the possibility that such modeling may be brought to bear on policy decisions. To that end, the context in which the model outputs will be used is highly relevant to modeling decisions. Policy-making needs are context specific; and in the case of modeling health co-benefits, model parameters may differ based on how health care delivery and public health costs are borne across sectors (e.g., how care is funded and handled at various levels of government). In developing their models and presenting their findings, researchers need to work with policy makers from the outset to ensure that the questions asked and analyses conducted are policy relevant.

A number of initiatives are underway that can serve as blueprints for building closer links between researchers and policymakers, such as the World Health Organization (WHO) Evidence Informed Policy Network (EVIPNet) initiative (WHO 2011) and Regional East African Community Health Policy Initiative Project (REACH) in East Africa (East African Community 2011). Despite such precedents, questions remain as to how to address certain key decision support issues. In particular, questions remain regarding the most ethically, morally, and economically defensible approach to valuation of future human health and well-being; whether and how to use discount rates; and what tools are best for comparing disparate types of costs, benefits, and constraints.

*The role of discounting and the effect of different discount rates*. Discount rates are central to all decisions with long-term implications, including co-benefits analyses that account for multiple costs and benefits distributed over time (Ackerman et al. 2009; Smith and Haigler 2008). When modeling health co-benefits, the basic function of discount terms is to convert future health and climate consequences of a mitigation measure into their net present value by subjecting the stream of monetized benefits and costs to a discount rate. Several options for handling discounting include ignoring it altogether or selecting constant, variable, or multiple rates for different components.

Setting the discount rate to zero. Avoiding discounting when modeling health co-benefits is equivalent to selecting a zero rate, which equates mitigation benefits and costs experienced today with those experienced in the very distant future. This may lead to situations where the current generation makes excessive sacrifices to future generations (Lopez et al. 2006). A major reason for discounting future benefits and costs is the expectation that future generations will be better off economically than present generations (Maddison 2001). Yet given the limitations on future growth imposed by resource constraints, we may experience a period of near zero real economic growth. In that case, a discount rate of zero or close to it may be justified depending on the time period of analysis.

Setting the discount rate to a constant above zero. Setting a nonzero discount rate can have equally unacceptable consequences by making catastrophic outcomes in the distant future appear trivial at today’s decision point, potentially biasing decisions against the interests of future generations (McMichael and Campbell-Lendrum 2003). Moreover, there is no consensus as to which discount rate to use (Weitzman 2001). This is problematic because widely varying policy decisions can be defended depending on the particular rate selected, posing a major challenge for analysis. One approach is to use several plausible rates to identify policies that are robust to the choice of rate (Lopez et al. 2006; Markandya et al. 2009; McKinley et al. 2005). Yet because of the strong sensitivity to the discount rate chosen, few policies may indeed be robust, and the benefits or costs may differ by large factors. For instance, in a model examining low-carbon electricity generation scenarios achieved through different degrees of emissions trading, Markandya et al. (2009) found that when the discount rate applied to lost life-years was increased from 0% to 3%, the estimated health co-benefits of low-carbon electricity generation scenarios were reduced by about 50%.

Setting variable discount rates. Some argue that a declining discount rate, which attaches increasing weight to the welfare of future generations, better reflects empirical data on individual preferences and is in agreement with various theoretical results (Dasgupta 2001; Heal 1997; Newell and Pizer 2003; Pearce et al. 2003; Reinschmidt 2002; Weitzman 2001). Although full hyperbolic discounting has not been supported by policy makers, there is a move toward declining discount rates driven by the dynamic uncertainty of future events (Pearce et al. 2003). Declining discount rates imply, for example, discounting benefits and costs that occur over the next 30 years at one rate, followed by a lower rate for benefits and costs that occur over the following 30 years and so on.

As an alternative to explicit discounting, some efforts instead use time horizons for certain terms, producing the odd result where consequences (i.e., costs or benefits) of an emission are accrued only up to a point, after which additional costs are ignored (Smith and Haigler 2008). Some have argued that smooth annual discounting functions are more sensible than the step-functions implied by such time horizons, such as those used to express the warming “costs” of an emission (Smith and Haigler 2008). Others argue that the various components common to co-benefits modeling should be discounted at different rates (Brouwer et al. 2005; Gravelle and Smith 2001).

Discount rates and their associated assumptions should be explicitly addressed in co-benefits research. For a particular intervention with both climate and health effects, rates must be specified for the costs of intervention (U.S. dollars), the impact on the global climate (tCO_2_; tons of carbon dioxide and other climate-active equivalents), the health effects (DALYs or QALYs) and the monetized health benefits (U.S. dollars), as discussed by Smith and Haigler (2008). Where available, locally estimated discount rates that reflect the specific values of affected populations should ideally be used. But because these are rarely available, and because there is no consensus on the selection of universal rates, an alternative approach would be to present results using several rates, including 0% and 3%, preferred values used by policy makers. Examining the implications of declining rates (HM Treasury 2003) would also be worthwhile.

*Evaluating mitigation options using decision analysis*. Accounting for potential health impacts of mitigation strategies is important, but many impacts unrelated to health exist, and policy makers require that alternative mitigation strategies be evaluated on the basis of many criteria simultaneously (Konidari and Mavrakis 2007; Swart et al. 2003). Valuation methods capable of considering trade-offs among multiple cost and benefit criteria under uncertainty are thus more likely to be policy relevant. To that end, the quantitative information on health criteria must be considered alongside nonhealth criteria, including economic growth, environmental sustainability, political acceptability, cost and financing considerations, expediency, and equity issues. Each of these can in turn be divided into detailed subcriteria, resulting in a deep hierarchical structure that defies single-criterion analytical approaches. For example, a cost and financing criterion could have subcriteria that include implementation costs, health services costs from changes in disease burden, opportunity costs of capital or land, and so forth. The performance of a mitigation strategy is unlikely to be positive (or negative) across all such criteria, and comparing short-term performance on certain criteria to long-term performance can raise important ethical questions—such as how should policy makers treat a renewable energy strategy that lowers short-term economic growth (and is thus temporarily detrimental to health because of reduced employment), but increases net health over the long-term from reduced pollutant emissions? Other ethical questions are raised by the fact that multiple criteria can at times represent competing stakeholder interests, such as a policy substituting active transport for single-occupancy vehicle use that reduces health costs while also decreasing revenues in the automotive sector.

The importance of consistent summary measures. Decision makers manage considerable complexity in part by determining which criteria are most relevant. At the same time, having a few summary or principal measures that are used consistently to assess different strategies greatly improves comparability. For example, a common measure for evaluating and comparing health co-benefits across alternative mitigation strategies and across countries is the health burden (DALYs) avoided, expressed per unit population size and per MtCO_2_ saved (Smith and Haigler 2008). Another useful and widely used measure is the net cost per ton of GHG emissions reduced. Many of the relevant outcomes, including health impacts, can, in principle, be converted into a monetary cost (Creutzig and He 2009). These costs can then be added to, or netted out, from the direct costs of the mitigation measures, giving a net cost figure per ton reduced. In calculating the measure, analysts face the problems described above (e.g., discounting, uncertainty), but the resulting information, partial as it is and with all its qualifications, is useful in deciding where to allocate scarce resources. The direct costs of mitigation may be, for example, US$30/tCO_2_, but when health co-benefits are accounted for, the figure may drop substantially or even become negative (i.e., result in net savings).

Multicriteria decision analysis (MCDA). Several decision analytical methods can be used to compare and evaluate alternative mitigation options in terms of their health and nonhealth impacts. These include traditional cost–benefit and cost-effectiveness methods used for environmental interventions (Haller et al. 2007; Hutton 2008). Because the impacts of mitigation are often multidimensional, more complex measures—and analytical methods—are needed for evaluating trade-offs. MCDA approaches have been used for this purpose in some policy areas, and their application to climate change policies is gaining momentum (Bell et al. 2001, 2003; Benegas et al. 2009; De Bruin et al. 2009; Kueppers et al. 2004; Stalpers et al. 2008; Wilbanks 2005).

There are unresolved issues in the application of MCDA methods to valuation of mitigation strategies. Traditional MCDA assumes that all criteria are evaluated at the same point in time. When comparing mitigation strategies where health is one of the criteria, assigning a relative weight to the health co-benefits criterion can be difficult because the immediate reduction in hazardous exposures does not often produce immediate health benefits (Jarrett et al. 2012; Wilkinson et al. 2010) (Table 2). This time course can be very different from those of the impacts of other criteria. In addition, because uncertainty increases into the future, issues surrounding attitudes toward risk (in the presence of uncertainty) and time preference become intertwined, complicating discount rate choices (Traeger 2009).

*Strategies to extend the model domain and policy utility*. Future directions for modeling co-benefits include enhancing policy relevance, addressing policy resistance, and characterizing implementation (including diffusions of new behaviors and technical shifts). Literature in recent years with respect to policy relevance highlights the importance of iteration between scientists and policy makers in developing usable science (Dilling and Lemos 2011). The National Oceanic and Atmospheric Administration (NOAA) Regional Integrated Science and Assessments (RISA) program is an example focused on climate change adaptation. RISA works with diverse user communities to advance contextual understanding of adaptation policy and management decisions; to develop knowledge on impacts, vulnerabilities, and potential response options; and to facilitate decision support tool development (NOAA 2012). Such an approach also may be particularly well suited to facilitating mitigation policy decisions.

“The counterintuitive behavior of social systems” (Forrester 1971) or “policy resistance” arises when policies that affect complex, dynamic systems result in unexpected outcomes, such as antibiotic resistance as a result of aggressive infection control or increased wildfire severity as a result of fire suppression (Sterman 2000). Systems dynamics methods (Sterman 2006) alone or in concert with other approaches such as discrete event simulation (Brailsford et al. 2010) can increase the likelihood of effective policy formulation (Thompson and Tebbens 2008) by addressing feedback loops that affect policy resistance. Many health co-benefit analyses characterize the health impacts of societal changes, such as widespread adoption of active transport policies or significant shifts in consumption of animal products, without a detailed consideration of how implementation might occur (e.g., Friel et al. 2009; Woodcock et al. 2009). Approaches such as agent-based modeling can help characterize diffusions of such innovations within populations and the role of organizations in catalyzing and maintaining significant policy shifts (Bonabeau 2002).

## Conclusions and Recommendations

Estimating the health impacts of GHG mitigation strategies is a complex process that brings together disparate disciplines. Because all models are simplifications that involve assumptions, are subject to many uncertainties, and capture a subset of interactions, modeling health co-benefits requires systematic consideration of the suitability of model assumptions, of what should be included and excluded from the model framework, and how uncertainty should be treated. The ultimate goal of modeling is policy utility, and it is important for modelers to iteratively engage policy makers actively in their work. Despite the challenges, there is a great need for information on the health implications of mitigation strategies, particularly given the urgency of bringing mitigation strategies into practice and the early accrual of ancillary health impacts of these strategies. Here we have reviewed some of the challenges and controversies in modeling health co-benefits and co-harms, and some approaches to increase their utility. Recommendations to improve such models include the following:

Modeling health co-benefits should be done in concert with policy makers from the start, and should focus on potentially feasible interventions based on policy-maker consultation; identification of policy-relevant outcomes; and incorporation, where needed, of methods to evaluate potential policy resistance. Model scoping should include consultation with policy makers and scientists from a range of disciplines to ensure that a full complement of potential impact pathways is considered. Focusing on domains and channels wherein modeling was used to affect policy may increase the potential utility of modeling efforts.
Initial stages of analysis should identify the full range of potential positive and negative pathways to health impacts within predefined boundaries, as well as the critical uncertainties in these causal pathways, while making explicit the criteria used to determine which exposure–outcome relationships are included in the model. The assessment of the strength of evidence for exposure–outcome relationships and parameters should use systematic review (Moher et al. 2009) and consensus methods (Guyatt et al. 2008).
The period over which the mitigation and health impacts are analyzed must be carefully assessed, both in relation to the time course between implementation of mitigation and consequent impacts, and in relation to time preferences for specific outcomes and the associated choice of discount rates. At a minimum, valuation estimates should be presented using a range of fixed discount rates including 0% and 3%, and consideration should be given to estimates using declining rates over time.
Uncertainty in modeling results should be characterized explicitly, using quantitative and qualitative methods as appropriate. Both parametric and structural uncertainties should be considered, and at a minimum, single (and when possible multivariate) deterministic sensitivity analyses should be carried out.
Scientists modeling health co-benefits should explicitly consider consulting with or including decision analysis experts to ensure that the results are useful in formal decision analysis processes. Such collaboration should be initiated at the inception of the modeling effort and should anticipate the ultimate application of the modeling results.

By improving the quality and rigor of health co-benefits analyses, critical decisions regarding climate mitigation strategies can be informed by health impact estimates, aiding policy makers in their efforts to maximize GHG mitigation potential while simultaneously improving health.

## Supplemental Material

(237 KB) PDF
