# Supplementary material for: Estimating the Health Effects of Greenhouse Gas Mitigation Strategies: Addressing Parametric, Model, and Valuation Challenges
Source: Environ Health Perspect. 2014 Feb 28;122(5):447–55. doi: 10.1289/ehp.1306744 (PMC4014758; doi:10.1289/ehp.1306744)
Supplement: (237 KB) PDF [file ehp.1306744.s001.pdf]

Environmental Health Perspectives doi:10.1289/ehp.1306744

Supplemental Material

Estimating the Health Effects of Greenhouse Gas Mitigation Strategies: Addressing Parametric, Model, and Valuation Challenges

Justin V. Remais, Jeremy J. Hess, Kristie L. Ebi, Anil Markandya, John M. Balbus, Paul Wilkinson, Andy Haines, and Zaid Chalabi

Table of Contents **Page**

Table S1 2

References 5

Table S1. Select literature providing quantitative estimates of health co-benefits of various climate change mitigation strategies. Each row refers specifically to the paper in the first cell and the outcomes, baseline data, scenarios, models, parameters, and results for the analyses its authors presented; see the primary publications for additional details.

Topic, citation	Mitigation policy (-ies)	Primary health co-benefits quantified	Baseline	Scenario assumptions	Model parameters	Cross-cutting issues	Estimated reduction in disease burden[§]	Major uncertainties
**Urban land transport (Grabow et al. 2012)**	Reduced rates of reliance on automobiles for short trips via replacement by active transport (walking and cycling) in the Midwestern region of the US.	Changes in air quality (reductions in ozone and $PM_{2.5}$) associated with respiratory and cardiovascular outcomes, lost productivity, and mortality, as well as increases in cardiovascular fitness from replacing 50% of short trips with cycling.	Air quality and vehicular travel data from surveys administered in 1995 and modeled emissions data from 2004.	Replacement of all short (≤ 4 km) automobile trips with active transport, 50% of which were by bicycle.	CR functions for $PM_{2.5}$ from US EPA regulatory impact analysis, for ozone from National Ambient Air Quality Standard, and for health impact of cycling from WHO tool	Reductions in emissions, including GHG emissions, from decreased automobile use; increased cardiovascular fitness from reduced emissions and increased physical activity; increased injuries from increased cycling activity.	1,295 deaths per year and savings of US$8 billion per year from improved air quality and improved cardiovascular health	How policy would be enacted and impacts of incentives to promote decreased reliance on automobiles for short trips; costs associated with infrastructure changes needed to support shift in cycling and pedestrian traffic
**Food and agriculture (Friel et al. 2009)**	Reduced rates of per capita saturated fat animal product consumption with replacement by polyunsaturated fat of plant origin in UK and in the city of São Paulo, Brazil	Reductions in morbidity and mortality associated with cardiovascular disease (ischemic heart disease [IHD] and stroke)	2009 rates of animal product consumption and associated cardiovascular disease burden	Reductions in emissions from technological change is insufficient to achieve desired 50% reduction in GHG emissions so 30% reduction in animal product consumption was assumed	RR reductions from systematic review of studies of dietary fat consumption and cardiovascular disease	Differences in GHG emissions according to whether ruminants are range or grain fed; potential for land use change, particularly deforestation, to contribute to emissions, depending on country	2900 (UK); 2200 (São Paulo , Brazil)	Trends in IHD burden and mortality, replacement of saturated fat from animal sources by polyunsaturated fats from plant sources

Topic, citation	Mitigation policy (-ies)	Primary health co-benefits quantified	Baseline	Scenario assumptions	Model parameters	Cross-cutting issues	Estimated reduction in disease burden[§]	Major uncertainties
**Health, agricultural, and economic impacts of tighter vehicle emissions standards (Shindell et al. 2011)**	Adoption of European vehicle emissions standards in developing countries compared with business as usual	Reductions in a suite of air pollutants emitted from on-road vehicles and related impacts on mortality and crop yields	2010 population and policy environment with full implementation of planned controls to 2050	Emissions standards feasible in various regions decided by expert opinion; no emissions uncertainties were included	Reported CRs for ozone and particulates from American Cancer Society cohorts and crop estimates based on published estimates	Impacts on agricultural production not included in most other assessments of mitigation strategies; crop impacts in some regions depend on crop mixtures	120,000-180,000 avoided annual air-pollution related deaths; 6.1-19.7 million metric tons of annual avoided crop losses	Tighter emissions standards will reduce short-term climate forcing but not reduce long-term climate change.
**Low-carbon electricity generation (Markandya et al. 2009)**	50% reduction in GHGs by 2050 in EU, India and China, achieved through different degrees of emissions trading	Reductions in fine particulate matter ($PM_{2.5}$) exposures, leading to reduced rates of cardiorespiratory disease, cancer, and lower respiratory tract infections	2009 mix of energy production methods and substrates , with associated GHG and particulate emissions	One scenario with global emissions trading, one with partial trading; scenarios compared with BAU	Socio economic and health impacts models from prior studies	Benefits depend on assumptions about the contributions of different sources of low carbon electricity	-100 years of life lost per million population (YLL) (EU); -550 YLL (China); -1500 YLL(India)	Heavy dependence on CCS, nuclear, and renewable; health effects of this mix of energy sources are not clear and in some cases may be subject to low-probability, high impact events
**Household energy (Wilkinson et al. 2009)**	Multipronged strategy to reduce GHG emissions from homes in UK; introduction of 150 million low-emission cookstoves in India	Improved household air quality and temperature regulation (UK) and improved household air quality and decreased mortality from ALRIs, COPD, and IHD (India)	2009 levels of weatherization and associated household temperature and household air quality exposures	Multiple potential interventions in UK with instantaneous change; introduction of 150 m low-emission cookstoves in India at 15 m stoves per year x 10 years	Derived from case studies in the UK and India	Technical improvements and impact on mitigation costs; fuel availability and costs; scenarios very ambitious but technically feasible	850 (UK); 12500 (India)	Dampened emissions reductions from efficiency gains as consumption rates grow and household wealth accrues; extent of penetration of low carbon electricity generation to replace fossil fuels for home heating

Topic, citation	Mitigation policy (-ies)	Primary health co-benefits quantified	Baseline	Scenario assumptions	Model parameters	Cross-cutting issues	Estimated reduction in disease burden[§]	Major uncertainties
**Urban land transport (Woodcock et al. 2009)**	Increased active travel and low emission vehicles in London and Delhi	Physical activity changes leading to reductions in IHD, diabetes, some cancers, dementia, and depression; benefits net of adverse impacts from increased road traffic injury rates in London, overall injury rates reduced in Delhi compared with BAU	2009 transport mix and practices in London and BAU evolution of transport policies in Delhi	Increased cycling and walking reduced private car use, increases in low emission vehicles, in various combinations	Case studies using data from London and Delhi	Need for city-specific data about transport patterns, physical activity levels and PM levels	7400 (UK); 13000 (Delhi, India)	Feasibility of radical reductions in car use, penetration of electric and other low emission vehicles; increases in injury rates
**Black carbon and methane emissions reductions (Anenberg et al. 2012)**	Global reductions in black carbon and methane emissions	Reductions in premature $PM_{2.5}$- and ozone-related mortality via a suite of different emissions reduction policy scenarios	Baseline 2005 emissions for the globe	BAU, BAU and adopted but not yet implemented measures, and three different, increasingly stringent scenarios for reducing black carbon and methane emissions, fully implemented and impacts on pollutant concentrations fully realized by 2030.	CR functions for $PM_{2.5}$ and ozone derived from American Cancer Society cohort studies	Meteorology and climatology held constant; impacts of mitigation measures on economic productivity and associated health impacts not included; changes in relative prevalence of infectious and chronic diseases not included	0.6-4.4 million deaths/year avoided for reduction in $PM_{2.5}$ and 0.04-0.52 million deaths/year globally for ozone	Benefits are underestimated as indoor particulate exposures are not included and spatial resolution limited application of outdoor exposure estimates.

Abbreviations: BAU: Business as usual; **CCS:** Carbon capture and storage; **GHG:** Greenhouse gas; **IHD:** Ischemic heart disease; **m**: million; **PM:** particulate matter. Note: the variables discussed here are the major ones for each study; this is necessarily a distillation of the complex modeling efforts that were described in each of these studies. [§]DALYs per million population unless otherwise noted.

References

Anenberg SC, Schwartz J, Shindell D, Amann M, Faluvegi G, Klimont Z, et al. 2012. Global air quality and health co-benefits of mitigating near-term climate change through methane and black carbon emission controls. Environ Health Perspect 120(6): 831-839.

Friel S, Dangour AD, Garnett T, Lock K, Chalabi Z, Roberts I, et al. 2009. Public health benefits of strategies to reduce greenhouse-gas emissions: food and agriculture. Lancet 374(9706): 2016-2025.

Grabow ML, Spak SN, Holloway T, Stone Jr B, Mednick AC, Patz JA. 2012. Air quality and exercise-related health benefits from reduced car travel in the midwestern United States. Environ Health Perspect 120(1): 68.

Markandya A, Armstrong BG, Hales S, Chiabai A, Criqui P, Mima S, et al. 2009. Public health benefits of strategies to reduce greenhouse-gas emissions: low-carbon electricity generation. Lancet 374(9706): 2006-2015.

Shindell D, Faluvegi G, Walsh M, Anenberg S, Van Dingenen R, Muller N, et al. 2011. Climate, health, agricultural and economic impacts of tighter vehicle-emission standards. Nat Clim Chang 1(1): 59-66.

Wilkinson P, Smith KR, Davies M, Adair H, Armstrong BG, Barrett M, et al. 2009. Public health benefits of strategies to reduce greenhouse-gas emissions: household energy. Lancet 374(9705): 1917-1929.

Woodcock J, Edwards P, Tonne C, Armstrong BG, Ashiru O, Banister D, et al. 2009. Public health benefits of strategies to reduce greenhouse-gas emissions: urban land transport. Lancet 374(9705): 1930-1943.
